# Evaluation of MTego and BG-Sentinel traps for malaria and arbovirus vector surveillance in Cabo Verde

**DOI:** 10.1371/journal.pgph.0007104

**Published:** 2026-08-18

**Authors:** Lara Ferrero Gómez, Adéritow Gonçalves, Hélio Rocha, Eliane Chantre, Eliane Gomes, Alba Reyes Concepción, Celivianne de Sousa, Suzineida Correia, Djeniffer Ramos, Adilson DePina, Marta Moreno, Jackie Cook

**Affiliations:** 1 Research Group on Tropical Diseases (GIDTPiaget), Life Sciences, Nature and Environment Unit, Jean Piaget University (UniPiaget), Praia, Cabo Verde; 2 Medical Entomology Laboratory, National Institute of Public Health, Praia, Cabo Verde; 3 Health Research Department, National Institute of Public Health, Praia, Cabo Verde; 4 Malaria Elimination Program, CCS-SIDA, Ministry of Health, Praia, Cabo Verde; 5 Department of Infection Biology, London School of Hygiene & Tropical Medicine, London, United Kingdom; 6 International Statistics and Epidemiology Group, London School of Hygiene & Tropical Medicine, London, United Kingdom; University of Cape Town, SOUTH AFRICA

## Abstract

Arboviral outbreaks are rising across Africa, a region that continues to face a substantial malaria burden. Cabo Verde remains vulnerable to the emergence and re-emergence of mosquito-borne diseases, including dengue, Zika, and malaria. In January 2024, the World Health Organization certified the country as malaria-free; however, the risk of reintroduction persists. Maintaining malaria-free status requires continuous robust epidemiological and entomological surveillance to prevent local transmission. Innovative vector monitoring and control tools that deliver accurate infestation data are essential in this context. We evaluated the performance of the MTego trap against the BGS traps routinely used in Cabo Verde applying a 4x4 Latin square design with four configurations: 1) BGS with BG Lure; 2) BGS with BG Lure plus CO_2_; 3) MTego traps with Clay Balls plus PM6 odour bait; and 4) MTego traps with Clay Balls, PM6 bait plus CO_2_. Sampling was conducted in Praia, before and after the 2023 rainy season. Trap effectiveness was assessed using negative binomial regression models. Across all configurations, 2,673 mosquitoes were collected. Mean nightly catches were highest for BGS traps baited with CO_2_: 20.4 (95% CI: 15.4-25.4), and lowest for MTego traps: 13.7 (95% CI: 10.0-17.5). Comparative analysis showed that MTego traps outperformed BGS traps in capturing *Anopheles arabiensis*, with CO_2_-supplemented MTego trap being the most effective configuration. BGS traps performed best for *Aedes aegypti* and *Culex pipiens* sensu lato. Species-specific behavioural patterns were observed, with higher nigh-time catches of *Cx pipiens* s.l. and *An. arabiensis*, alongside differences in gonotrophic state. This study highlights the superior performance of MTego traps for malaria surveillance in Cabo Verde.

## Introduction

It is estimated that 80% of the global population is at risk of at least one vector-borne diseases (VBDs) [1]. Mosquito-borne diseases alone threaten over 40% of the global population [2]. Arbovirus outbreaks are increasing across Africa a region already burdened by malaria. For instance, dengue is circulating in over 30 African countries. Between January 2024 and August 2025, nineteen African countries reported dengue outbreaks, with more than 85,000 confirmed cases [3–5]. Since 2023, a resurgence of chikungunya has been documented in West Africa, with over 83,000 confirmed cases across Senegal, Burkina Faso, Mali, Chad, Kenya, Ethiopia, Gambia, Guinea, and Mauritius [6].

Cabo Verde, an African archipelago in the Atlantic Ocean, is vulnerable to the emergence and re-emergence of VBDs due to its geographical location, climate conditions, and the effects of intense movement of people and goods [7]. On 12 January 2024, the World Health Organization (WHO) certified Cabo Verde as malaria-free [8]. Maintaining this status is a major challenge, requiring robust epidemiological and entomological surveillance systems to prevent secondary local transmission from imported malaria cases [9,10].

Dengue virus (DENV) has also been reported in the archipelago, with *Aedes aegypti* recorded in all the islands [11]. This species caused two major dengue epidemics, with the most recent occurring between November 2023 and March 2025, with over 17,000 confirmed cases and 8 deaths It was also implicated in the 2015–2016 Zika epidemic, with more than 7,500 confirmed cases and 18 associated cases of microcephaly [12–15]. Yellow fever, transmitted by *Ae. aegypti,* has likewise been reported in the islands [16]. *Culex pipiens* s.l. is also present in Cabo Verde and is a vector of West Nile virus (WNV) and Rift Valley fever virus (RVFV), both reported elsewhere in Africa [17]. To combat these diseases, Cabo Verde implemented a National Integrated Vector Control Program, based on awareness campaigns, elimination or treatment of potential breeding sites, and indoor pyrethroid spraying before and after rains [18].

WHO recommends integrated management of VBDs as a cost-effective strategy, with vector surveillance and monitoring as essential components [1,19]. One of the challenges facing vector control in Cabo Verde is optimizing adult mosquito monitoring tools, particularly for capturing anophelines in the context of malaria elimination. Currently, adult mosquito surveillance in Cabo Verde relies on Biogents-Sentinel (BGS) traps, mechanical aspiration, and CDC light traps, which the latter generally showing poor performance. Because adult traps are a key component of entomological surveillance programs in risk areas [20,21], there is an urgent need for more effective and sustainable traps for vector control in Cabo Verde, particularly for *An. arabiensis*. Effective traps for monitoring multiple species are essential for countries at risk of different VBDs [22]. Although the BGS trap performs well for *Ae. aegypti*, comparative studies of different trap types are needed, to characterize performance, particularly for adult *Anopheles* mosquitoes, to strengthen surveillance and prevent malaria reintroduction in Cabo Verde. In this context, in this study, the BGS trap was selected as the reference comparator because it is the tool most consistently used for operational surveillance of host-seeking mosquitoes in Cabo Verde, whereas the CDC light trap, despite its routine use, shows limited effectiveness in this setting and was therefore not included as an additional comparator.

This study evaluated the performance of the MTego traps (PreMal BV, Netherlands) [23], developed for capturing anophelines, against BGS traps (Biogents, Germany), the main tool routinely used in Cabo Verde for mosquito surveillance. The MTego trap is an odour-baited device that incorporates heat and moisture cues to enhance mosquito capture [24]. The study specifically compared trap performance for anophelines and non-anophelines species in an urban setting in Cabo Verde.

## Materials and methods

### Ethics statement

All mosquito collections were conducted within private properties. Owners were informed about the study activities, and collections were authorized through signed informed consent. No endangered or protected species were involved in the fieldwork. The materials employed (traps and related equipment) posed no health risk to either researchers or property owners, and no vertebrate animals were harmed. Ethical approval for the project was granted by the Ethics Committee of the London School of Hygiene and Tropical Medicine (Ref. 29159) and by the National Ethics Committee for Health Research of Cabo Verde (Deliberation nº21/CNEPS/2023).

### Study area

Mosquito sampling was conducted in the city of Praia, the capital and main urban centre of Santiago Island, located on the western coast of Africa in the Atlantic Ocean (between latitude 14˚ and 18˚ N and longitude 22˚ and 26˚W). The climate is subtropical and dry, with an arid season lasting most of the year and a short rainy season from July to October. Annual rainfall ranges from 200 to 600 mm, and the average temperature is 25˚C [25]. Previous studies indicate that Santiago, where Praia is located, hosts the greatest mosquito species richness in Cabo Verde, including major vectors such as *Ae. aegypti*, *An. arabiensis* and members of the *Cx. pipiens* complex [26]. These species exhibit distinct ecological and behavioural patterns. *Ae. aegypti* is strongly associated with domestic artificial containers and is highly anthropophilic [11]; *An. arabiensis* breeds in temporary and semi-permanent outdoor habitats [10] and shows low human blood preference, feeding on multiple vertebrate hosts; and *Cx. pipiens* s.l. displays more opportunistic feeding behaviour across humans and domestic animals [27].

The study was conducted in Vale do Palmarejo, a neighbourhood of Praia, considered a risk zone for malaria and other mosquito-borne diseases. The area includes agricultural plots, domestic animals, multiple aquatic breeding sites, and households with piped water but recurrent sanitation problems [28]. Socioeconomic status is not formally classified at the sub‑municipal level in Cabo Verde; therefore, this neighbourhood cannot be categorized as low or high socioeconomic status based on official indicators. Long-term entomological surveillance and field observations instead suggest heterogeneous environmental and residential conditions rather than a clearly defined socioeconomic profile.

### Study design

**Fig 1:**
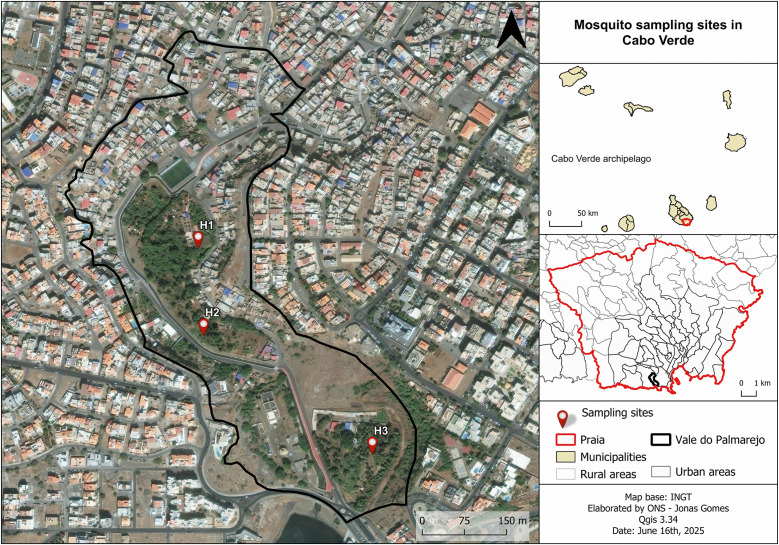
Location of the three sampling sites (H1, H2, and H3) in Vale do Palmarejo, city of Praia.

To evaluate the four-trap survey design, three privately owned agricultural properties were selected as sampling sites: (H1: 14°54’33.3"N 23°31’22.8"W; H2: 14°54’28.2"N 23°31’22.4"W; and H3:14°54’21.0"N 23°31’12.4"W) (Fig 1).

Selection criteria included the historically known presence of *Anopheles* species, proximity to mosquito breeding sites, accessibility, availability of power supply, and their strategic distribution along the entire length of Vale do Palmarejo. In addition, traps were placed at least 25 m apart and sampling sites at least 200 m apart to minimize potential interference from odour plumes emitted by neighbouring traps.

### Mosquito sampling and identification

**Fig 2:**
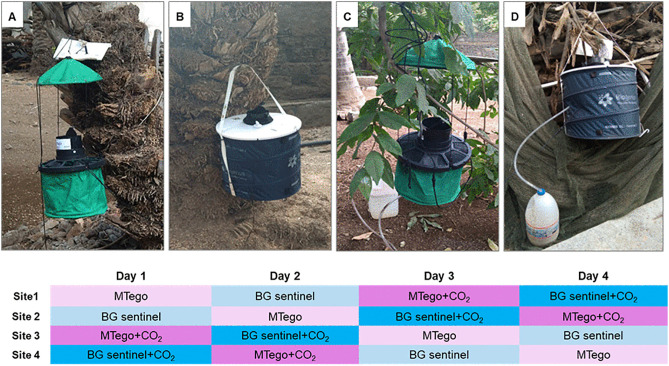
Type of traps and collection design. **A) MTego trap; B) BGS; C) MTego + CO**_**2**_**; D) BGS + CO**_**2**_. Traps and collection cycle, based on a 4x4 Latin square design, of mosquitoes captured in Vale do Palmarejo, city of Praia, Cabo Verde. Traps rotated every 24 hours, collecting mosquitoes every 12 hours from 7 a.m. and 7 p.m. A complete capture cycle comprised 4 days with 4 replicates per cycle (16 days in total).

Two adult mosquito surveys were conducted between June and December 2023 in the municipality of Praia, one prior the rainy season (13 June to 7 July 2023) and another following the rainy season (31 October to 1 December 2023). Four variations of two mosquito traps were deployed in a 4x4 Latin square design (Fig 2): 1) BGS with BG Lure; 2) BGS with BG Lure plus CO_2_; 3) MTego trap with clay balls and PM6 odour attractant; and 4) MTego trap with clay balls, PM6 odour attractant and CO_2_. In each season, the sampling period lasted approximately one month, corresponding to the time required to complete the four rotation cycles of the 4 × 4 Latin square design. Each cycle involved repositioning the four trap types so that every trap occupied each sampling position exactly once, ensuring balanced spatial and temporal replication under field conditions.

The MTego is an adult mosquito trap that operates with a suction mechanism and mimics human characteristics to attract mosquitoes through a PM6 scent bag, optimized to enhance Anopheles captures. An innovative feature of this trap is the use of clay balls, which, once moistened with water, act as a CO_2_ surrogate by stimulating host-seeking behaviour, thereby eliminating the need for CO_2_ tanks. Carbon dioxide was also incorporated into our design following the fermentation-based method described by Smallegange *et al*. (2010) [29], with minor modifications: 30 g of dry yeast, 300 g of white sugar, and 3L of tap water mixed in a 5L plastic bottle connected to the trap via silicone tubing. These proportions were adjusted based on preliminary field observations in Praia to ensure stable CO_2_ release under local operational conditions. This approach provides a practical alternative for generating a continuous CO_2_ plume suitable for mosquito host-seeking studies [29].

Traps with or without CO_2_ were arranged in a crosswise pattern across four points (A, B, C, and D) and rotated sequentially following a latin square design over four consecutive nights per cycle at the three sampling sites (H1, H2, and H3). Each trap was placed at least 25 m apart and rotated every 24 hours, with 12 hours collection periods (diurnal: 7 a.m.-7 p.m.; nocturnal: 7 p.m.-7 a.m.) (Fig 2). This design was applied in both mosquito sampling surveys. Collected specimens were transported either to the Entomology Laboratory of the Tropical Diseases Research Group of Jean Piaget University (GIDTPiaget) for nocturnal collections or to the Medical Entomology Laboratory of the National Institute of Public Health for diurnal collections in Praia. Mosquitoes were manually counted and morphologically identified, using taxonomic keys [30–33] and the female gonotrophic state was assessed [34].

Molecular identification was performed for specimens with inconclusive morphological results. DNA was extracted [35], and its quantity and purity were assessed using a SimpliNano micro-volume spectrophotometer. Differentiation of *An. arabiensis* was achieved by PCR using adapted published methods [36] with universal and *An. arabiensis* specific primers (UN: 5′- GTG TGC CCC TTC CTC GAT GT -3′; AR: 5′- AAG TGT CCT TCT CCA TCC TA -3′). Amplified DNA was separated on a 1.5% agarose gel stained with Safe-Green and documented using the VWR SMART5 Gel Documentation System.

### Climatic data

Air temperature and relative humidity were measured daily during both study periods using a Bresser portable weather station installed at one of the three agriculture properties. Wind speed was recorded during the first period, but wind direction was not measured. A malfunction of the station during the post‑rainfall period prevented consistent wind‑speed recording; therefore, wind‑related variables were excluded from the analyses. Trap placement did not consider wind direction and was determined solely by the minimum separation distance required to avoid interference (>25 m), within the 4 × 4 Latin square rotation scheme applied in the study.

### Statistical analysis

Descriptive statistics were used to summarize the climatic data (S1 Data) and the distribution of captured mosquitoes (S2 Data), presented in tables and figures generated in RStudio (2026.01.1 + 403, Apple Blossom). Negative binomial regression was applied due to the high frequency of zero catches. One model included four trap types (BGS, BGS with CO_2_, MTego, MTego with CO_2_) as fixed effects, while adjusting for season, trap position (A, B, C, D), and collection site. Because sampling locations were visited repeatedly over time, “collection site” was included as an adjustment factor to account for the repeated measurements at each location. Mixed‑effects specifications with random intercepts for sampling location were also evaluated to capture potential clustering; however, these models were unstable due to the relatively small number of observations per site–position–period combination and did not improve model fit according to AIC/BIC. Therefore, all predictors were treated as fixed effects. The BGS without CO_2_ was considered the reference category, as it is the trap most routinely used in national mosquito surveillance activities in Cabo Verde. This choice enables direct comparison of density ratio among the four methods. A second model included trap type as a fixed effect with an interaction for CO_2_ to evaluate impact of trap performance. This model was adjusted for the same confounders as the first. Statistical significance was defined at a 5% threshold (p < 0.05).

## Results

A total of 2,673 mosquitoes were captured across 874 trap deployments during the two surveys: 18.6% (n = 498) in MTego traps, 27.2% (n = 726) in MTego + CO_2_ traps, 25.9% (n = 691) in BGS traps and 28.4% (n = 758) in BGS + CO_2_ traps. Approximately one-third of daytime traps deployment (n = 261; 30.2%) did not collect any mosquitoes. Overall, *Aedes aegypti* and *Cx. pipiens* s.l. represented the majority of captures, with mean nightly densities of 33.8 (95% CI, 27.5-40.2) and 29.7 (95% CI, 25.1-34.2), respectively, whereas Anopheles spp. were collected at markedly lower densities (7.7; 95% CI, 3.2-12.2). *Anopheles arabiensis* was predominantly collected (96.7%, 263/272) during nocturnal periods (7 p.m.-7 a.m.), and following the rainy season (94.5%, 257/272). In contrast, most *Ae. aegypti* and *Cx. pipiens* s.l. were collected before the rainy season (64.3%, 840/1,307; and 83.8%, 910/1,087, respectively), with a high proportion of *Cx. pipiens* s.l. caught at night (94.2%, 1,023/1086). *Aedes aegypti* was collected during both diurnal and nocturnal periods (42.9%, 561/1,307; and 57.1%, 746/1,307, respectively). A greater proportion of *Ae. aegypti* specimens were females (77.9%, 1,018/1,307), of which 23.7% (241/1,018) were gravid and 73.4%, (746/1,018) unfed (Table 1).

**Table 1:** Summary of mosquito collections by species.

Variable	*An. arabiensis* N = 272	*An. pretoriensis* N = 7	*Ae. aegypti* N = 1,307	*Cx. pipiens* s.l. N = 1,086
**Trap type**				
BGS	61 (22.4%)	2 (28.6%)	337 (25.8%)	290 (26.7%)
BGS + CO_2_	59 (21.7%)	4 (57.1%)	389 (29.8%)	306 (28.2%)
MTego	65 (23.9%)	1 (14.3%)	187 (14.3%)	245 (22.6%)
MTego + CO_2_	87 (32.0%)	0 (0.0%)	394 (30.1%)	245 (22.6%)
**Sex**				
Female	148 (54.4%)	4 (57.1%)	1,018 (77.9%)	532 (49.0%)
Male	124 (45.6%)	3 (42.9%)	289 (22.1%)	554 (51.0%)
**Collection period**				
7 p.m. to 7 a.m.	263 (96.7%)	7 (100.0%)	746 (57.1%)	1,023 (94.2%)
7 a.m. to 7 p.m.	9 (3.3%)	0 (0.0%)	561 (42.9%)	63 (5.8%)
**Season**				
Before rainy season	15 (5.5%)	0 (0.0%)	840 (64.3%)	910 (83.8%)
After rainy season	257 (94.5%)	7 (100.0%)	467 (35.7%)	176 (16.2%)
Gonotrophic state*				
Freshly fed	41 (27.7%)	4 (100.0%)	11 (1.1%)	39 (7.3%)
Semigravid	6 (4.1%)	0 (0.0%)	19 (1.9%)	40 (7.5%)
Gravid	1 (0.7%)	0 (0.0%)	241 (23.7%)	224 (42.1%)
Unfed	100 (67.6%)	0 (0.0%)	746 (73.4%)	229 (43.0%)

One specimen of *Anopheles spp.* could not be differentiated and was omitted from the table.

*Only applicable to females.

Descriptive statistics were applied to summarize climatic variables (S1 Data) and the overall distribution of captured mosquitoes (S2 Data). Table 1 presents aggregated counts to describing species composition and distribution across trap types, sex, collection periods, seasons, and gonotrophic states. As these values do not represent replicate‑level observations, measures of central tendency or dispersion (e.g., medians or 95% confidence intervals) are not applicable. Inferential analyses based on replicate‑level data are reported in the regression results, where density ratios and their 95% confidence intervals were estimated using negative binomial models adjusted for season, trap position, and collection site

With respect to temperature and humidity during the study period, no significant differences were detected. The mean air temperature was 27.9 °C (95% CI, 27.8-30.0) and remained relatively stable across the two phases, with mean values of 28.2 °C (95% CI, 27.9-28.5) before rainfall and 27.4 °C (95% CI, 26.9-27.9) after rainfall. Diurnal fluctuations were minimal, averaging approximately 2 °C during the day and 2.5 °C at night. The mean relative humidity was 64.9% (95% CI, 63.2-66.6), with a maximum variation of 12.8% between phases, indicating drier conditions following rainfall.

**Fig 3:**
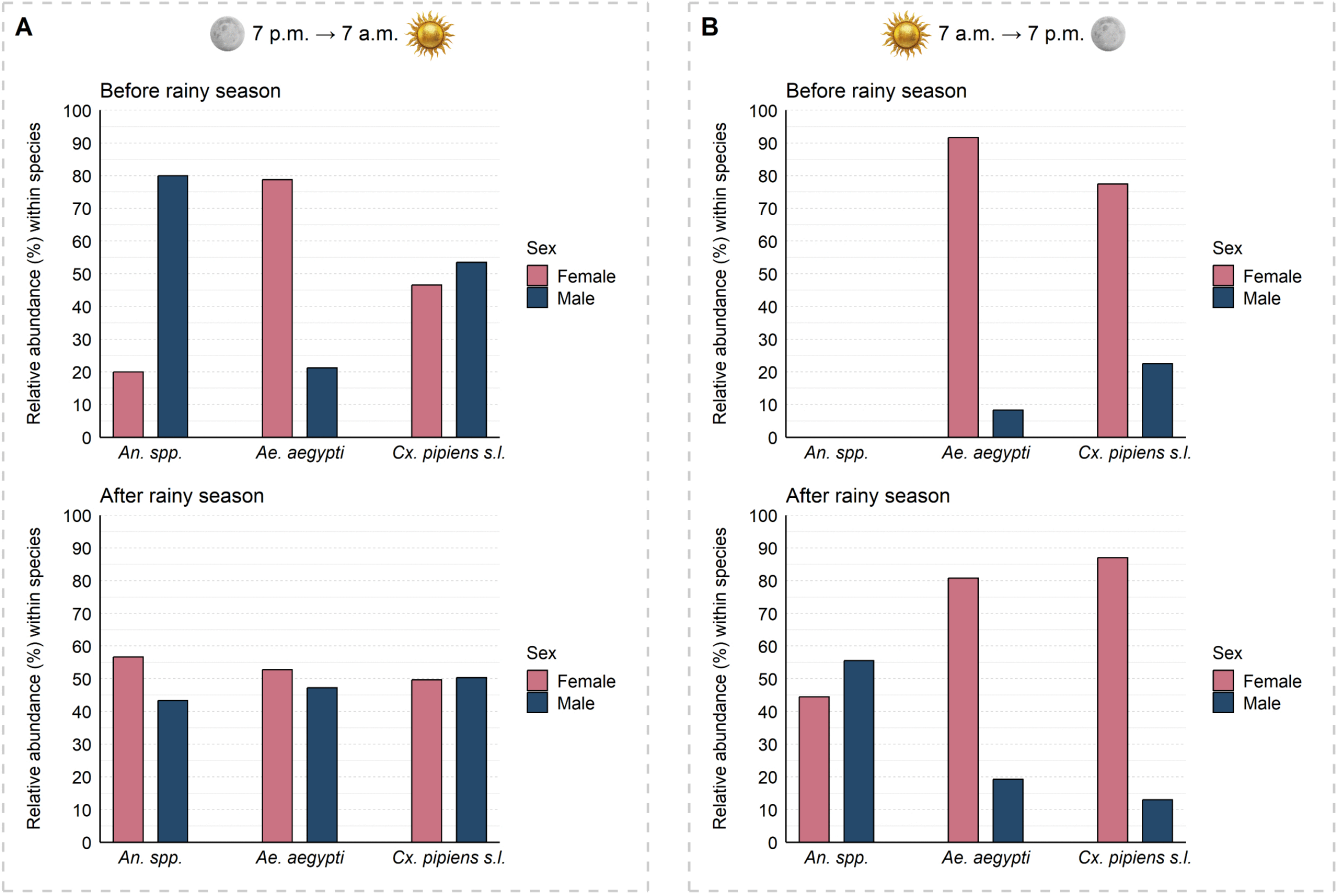
Relative abundance (%) of mosquito species by sex before and after the rainy season across diel periods. (A) Night-to-morning; (B) Morning-to-night.

Fig 3 illustrates the relative abundance of male and female mosquitoes within species according to diel period (night-to-morning and morning-to-night) and season (before and after the rainy season). For visualization, *An. arabiensis*, *An. pretoriensis*, and one *Anopheles* spp. were grouped under *Anopheles* spp.

Before the rainy season, distinct sex-specific patterns were observed. During nocturnal collections, *Anopheles spp*. were predominantly male, whereas *Aedes aegypti* exhibited a pronounced female bias. *Culex pipiens* s.l. showed a more balanced sex distribution at night. In contrast, daytime collections before the rainy season were strongly dominated by females of *Ae. aegypti* and *Cx. pipiens* s.l., while *Anopheles* spp. was virtually absent. Following the rainy season, nocturnal collections revealed a more balanced sex ratio for Anopheles spp., *Cx. pipiens* s.l., and *Ae. aegypti*. During daytime and after the rainy season, females predominated in *Ae. aegypti* and *Cx. pipiens* s.l., whereas Anopheles spp. displayed a higher proportion of males.

Trap performance varied by species and trap type (Table 2). For analytical purposes, all anophelines collected were grouped under Anopheles spp.

**Table 2:** Female mosquito captures and trap efficiency estimates from negative binomial analysis.

Mosquito species	Trap type	Total catch	Mean catch per trap-night	Density ratio	95% CI^a^	^*c*^ *p-value*
*Anopheles* spp.	^b^BGS	22	0.69	1	Ref.	–
	BGS + CO_2_	30	0.94	1.4	0.8-2.5	0.308
	MTego	36	1.13	1.6	0.9-2.9	0.122
	MTego + CO_2_	64	2.00	3.0	1.7-5.1*	<0.001
*Aedes aegypti*	^b^BGS	286	8.94	1	Ref.	–
	BGS + CO_2_	312	9.75	1.2	0.7-1.8	0.539
	MTego	150	4.69	0.5	0.3-0.9*	0.009
	MTego + CO_2_	270	8.44	0.9	0.5-1.3	0.501
*Culex pipiens* s.l.	^b^BGS	177	5.53	1	Ref.	–
	BGS + CO_2_	157	4.91	0.9	0.5-1.5	0.668
	MTego	116	3.63	0.7	0.4-1.2	0.205
	MTego + CO_2_	82	2.56	0.5	0.3-0.9*	0.010

a CI: Confidence interval. 95% confidence intervals were estimated using a negative binomial model to evaluate the effectiveness of the traps with trap type included as a fixed effect, whilst controlling for season and location. ^b^The BGS trap was used as the reference category for comparisons.

c Statistical significance was determined based on *p-values* less than 0.05, and significant results are indicated with an asterisk (*).

For Anopheles spp., the MTego + CO_2_ trap captured significantly more mosquitoes than the BGS trap (density ratio = 3.0; 95% CI: 1.7-5.1; p < 0.001), while the BGS + CO_2_ trap did not provide strong evidence of increased catches (density ratio = 1.4; 95% CI: 0.8-2.5; p = 0.308) compared to the BGS trap without CO_2_. Similarly, *Aedes aegypti* captures in the BGS + CO_2_ trap were comparable to BGS alone (density ratio = 1.2; 95% CI: 0.7-1.8; p = 0.539), whereas the MTego trap alone showed significantly lower catches (density ratio = 0.5; 95% CI: 0.3-0.9; p = 0.0097) compared to BGS alone. For *Culex pipiens* s.l., the MTego + CO_2_ trap captured significantly fewer specimens than the BGS (density ratio = 0.5; 95% CI: 0.3-0.9; p = 0.010), and the addition of CO_2_ to the BGS trap did not increase catches (density ratio = 0.9; 95% CI: 0.5-1.5; p = 0.554). A direct comparison between MTego + CO_2_ and BGS + CO_2_ indicated that the MTego trap collected more than twice as many mosquitoes as the BGS trap (density ratio = 2.2; 95% CI: 1.3-3.7; p = 0.003), after adjusting for confounders.

The interaction between trap type and CO_2_ was not significant for *Anopheles spp.* (p = 0.436), *Ae. aegypti* (p = 0.315) or *Cx pipiens* s.l. (p = 0.526) indicating no evidence that the addition of CO_2_ bait produced differential effects depending on trap type. Carbon dioxide was associated with increased catches in MTego traps (density ratio = 1.9; 95% CI: 1.1-3.0; p = 0.012), whilst the effect was not statistically significant in BGS traps (density ratio = 1.4; 95% CI: 0.7-2.5; p = 0.308). For *Ae. aegypti*, CO_2_ was likewise associated with increased catches in MTego traps (density ratio = 1.6; 95% CI: 1.0-2.6; p = 0.048), but not in BGS traps (density ratio = 1.2; 95% CI: 0.7-1.8; p = 0.539). For *Cx. pipiens* s.l, there was no evidence that CO_2_ increased catches in either trap type.

**Fig 4:**
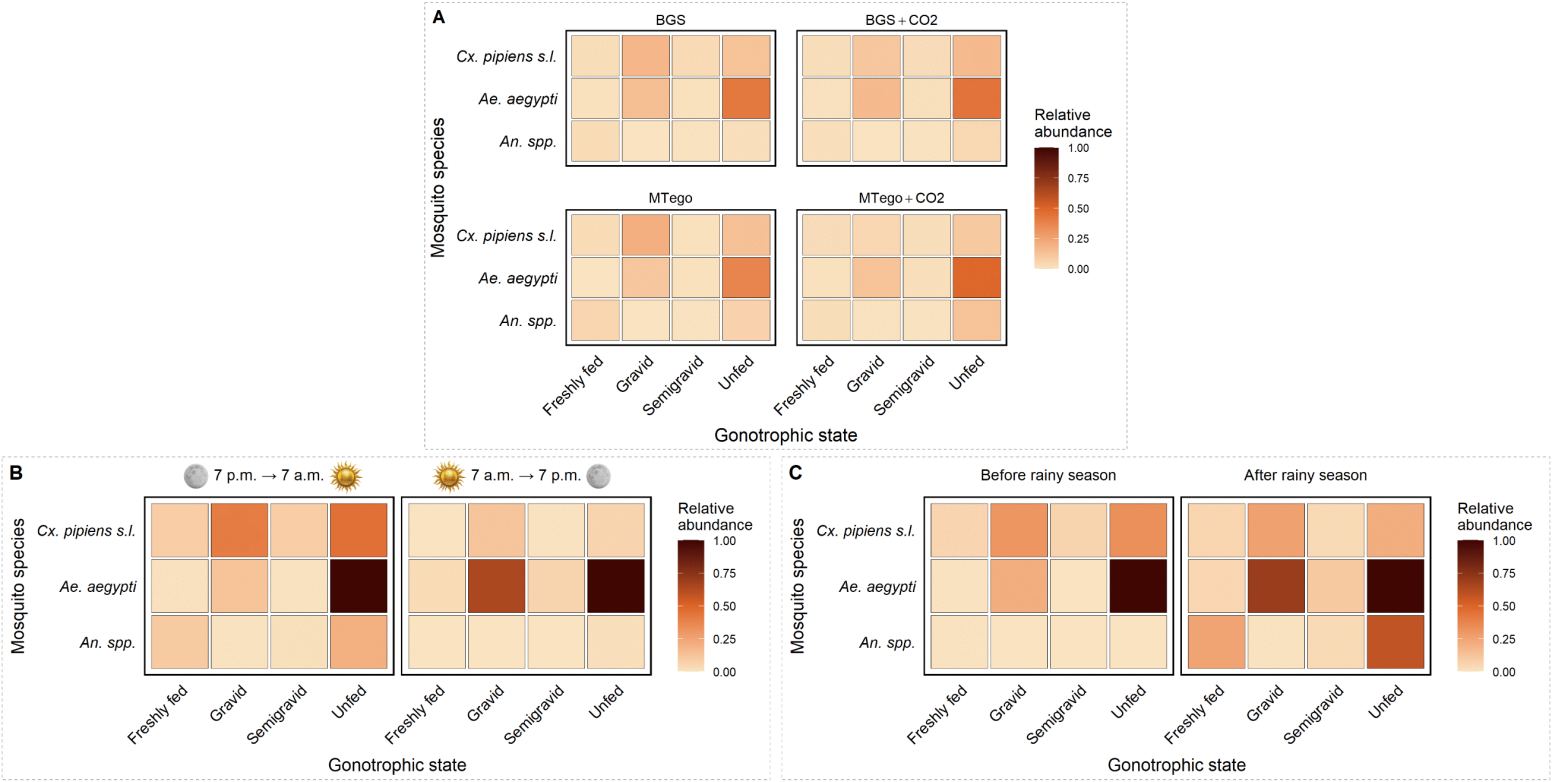
Physiological variation (freshly fed, gravid, semigravid, and unfed) in female mosquito species across trap type (A), diel period (B), and season (C).

Composite heatmaps (Fig 4) illustrate the relative abundance of freshly fed, gravid, semigravid, and unfed female mosquitoes according to trap type, diel period and season. For this analysis, all anophelines were grouped.

The gonotrophic profile of *Ae. aegypti* was similar across all four traps (Fig 4A), characterized by a high abundance of unfed mosquitoes and a moderate abundance of gravid individuals. Gravid Anopheles spp. mosquitoes were nearly absent across all trap types. Unfed Anopheles spp. were captured in greater numbers by traps supplemented with CO_2_ (MTego, 82.8% (n = 53); BGS, 66.7% (n = 20)), with most specimens collected alongside freshly fed mosquitoes. In traps without CO_2_, an opposite pattern was observed, particularly with MTego (56.6%, n = 12), where freshly fed *An. arabiensis* were captured in greater or comparable numbers relative to unfed specimens For *Cx. pipiens* s.l. more unfed individuals were collected in traps containing CO_2_, whereas gravid mosquitoes were more abundant in traps without CO_2_ supplementation. A distinct pattern was observed for female *Anopheles. spp*. mosquitoes, with nearly all captures occurring at night (Fig 4B). Most specimens were either unfed (n = 96) or recently fed (n = 45). The number of unfed *Ae. aegypti* captured during the nocturnal period exceeded daytime collections, representing 62.5% (466/746) of all female *Ae. aegypti* collected. Gravid *Ae. aegypti* were more frequently captured during the day, in contrast to *Cx. pipiens* s.l., for which gravid individuals were more abundant at night. Higher numbers of unfed and gravid *Ae. aegypti* and *Cx. pipiens* s.l. specimens were collected before the rainy season (Fig 4C). For *Ae. aegypti,* the proportion of gravid to unfed specimens before the rainy season was relatively low (122 (gravid)/576 (unfed) = 0.212).

## Discussion

Novel, sustainable, and effective tools for monitoring the primary mosquito vectors responsible for the transmission of major vector-borne diseases in Cabo Verde (malaria, dengue, and Zika) are essential to prevent the reintroduction of malaria and to establish robust preventative systems for other mosquito-borne diseases. This study evaluated the effectiveness of four adult trap types for mosquito sampling in Praia, specifically in Vale do Palmarejo neighbourhood, a recognized malaria hotspot.

Trap performance varied according to mosquito species (MTego and BGS, with or without CO_2_ supplementation from a yeast-fermented solution). MTego traps, particularly when baited with CO_2_, greater numbers of female *An. arabiensis* compared to the BGS. In contrast, more *Cx. pipiens* s.l. and *Ae. aegypti* females were collected using BGS traps. These findings underscore the suitability of the MTego trap for monitoring *An. arabiensis* in Cabo Verde and provide insights for optimizing vector surveillance strategies in Praia, with trap selection tailored to the surveillance objective (malaria vs. arboviruses) [28]. Our results differ from a semi-field study [23], which reported similar performance of MTego and BGS traps for *Ae. aegypti* and a lower performance of the MTego in sampling *An. arabiensis*. This discrepancy highlights the variable performance of mosquito traps across ecological contexts. Methodological differences may also contribute to these variations, such as semi-field versus field conditions and the use of laboratory-reared versus wild mosquito populations. An additional factor that may explain the divergence is the CO_2_ supplementation employed in the present study. As CO_2_ is both an activator and attractant for several mosquito species [37,38], its inclusion may have enhanced the relative attractiveness of the traps, limiting direct comparability with evaluations conducted without CO_2_. This should be taken into consideration when interpreting differences among studies.

Beyond performance considerations, the operational cost structure of the traps also differs. MTego traps require proprietary attractant cartridges that must be periodically replaced, whereas BGS traps rely on BG‑Lure sachets and, when applicable, CO_2_ supplementation. In our field context, CO_2_ was generated using a low‑cost yeast–sugar fermentation system, but this approach is labor‑intensive and requires daily preparation and handling. Although exact monetary costs vary across suppliers and regions, MTego traps generally involve higher upfront and consumable expenses, whereas BGS traps combined with homemade CO_2_ represent a more economical but operationally demanding alternative. These cost differences are relevant for surveillance programs when selecting trap types according to budget, logistical capacity, and target vector species.

*Aedes aegypti* was the predominant species throughout the study period, followed by the *Cx. pipiens* complex. Both species were most abundant before the rainy season, with a marked decline in *Cx. pipiens* s.l. populations afterwards coinciding with the emergence of *An. arabiensis*. The higher density of *An. arabiensis* in Praia following the rainy season is consistent with findings from other studies [39,40] that link rainfall, temperature, and humidity with the proliferation of breeding sites for these species, as observed in the Vale do Palmarejo.

The high numbers of *Cx. pipiens* s.l. and *Ae. aegypti* recorded in our study are consistent with the high efficiency of BGS in sampling these two species [41,42], whereas Anopheles capture rates were comparatively lower under the same field conditions.

In our study, MTego traps supplemented with CO_2_ captured nearly twice as many female *An. arabiensis* as MTego traps without CO_2_, demonstrating the synergistic effect of CO_2_, combined with PM6 odour bait as a long-range mosquito attractant. Conversely, no significant differences were observed for the BGS trap. Similar findings were reported in semi-field studies using the anopheline scent bait MB5 + CO_2_ to capture *An. arabiensis* [43]. Although clay balls have been described as a potential CO_2_‑generating component [23,24], their specific contribution could not be assessed in our study because MTego configurations with and without clay balls were not directly compared. Therefore, any inference regarding their effect on mosquito captures is not supported by the available data.

Although the MTego trap showed promise for monitoring *An. arabiensis* in Cabo Verde, capture rates were lower than for *Ae. aegypti* and *Cx. pipiens* sensu lato. This may reflect either lower population densities or reduced trap effectiveness, as the PM6 odour bait was originally developed for *An. coluzzii* 4] and tested with *An. gambiae* s.s.*,* rather than *An. arabiensis* [43]. Evidence on the attractiveness of PM6 to *An. arabiensis* under field conditions remains limited, and further studies are needed to confirm this hypothesis. Additionally, the opportunistic feeding behaviour of *An. arabiensis*, with a human blood index of 0.40 in Praia and other sites on Santiago Island [27], may further influence trap performance.

BGS traps supplemented with CO_2_ performed better for *Ae. aegypti* and *Cx. pipiens* s.l, suggesting their suitability for surveillance of these species in Cabo Verde [44–46]. However, species-specific ecological adaptations, genetic traits, and environmental factors must be considered when evaluating trap performance in new ecological contexts [47–49].

Our study area was selected based on the strong spatial association between the presence of the malaria vector and reported cases in this area [39], likely influenced by environmental conditions such as agricultural areas and year-round water availability in a low-lying part of the city, near the dry stream by the sea. This area was specifically chosen to increase the likelihood of capturing *Anopheles* mosquitoes; however, this may limit the generalizability of our findings to other settings, in with different environmental contexts. We also deliberately targeted two seasons to capture differences between before and after rainy seasons, which were evident in the data. Nonetheless, this approach may also restrict generalisability to other seasonal conditions.

This study demonstrated that *An. arabiensis* and *Cx. pipiens* s.l. were predominantly captured during the night, consistent with their known nocturnal biting behaviour [50–53]. Evidence of nocturnal activity is particularly relevant for the evaluation and implementation of malaria control strategies based on human exposure patterns, such as indoor residual spraying [54]. and personal nighttime protection. *Aedes aegypti* showed a less defined pattern with a higher proportion of daytime catches.

Our findings confirm that seasonality influenced mosquito species composition. *Anopheles arabiensis* was mainly captured after the rainy season, whereas *Ae. aegypti* and *Cx. pipiens* s.l. were more abundant before the rainy season. These results highlight a potentially higher risk of malaria transmission in Cabo Verde at the end of the rainy season. Typically, increases in *An. arabiensis* density are associated with environmental factors such as availability of aquatic habitats, humidity, temperature, and wind speed [55–61]. In our study, higher *An. arabiensis* captures followed rainfall aligning with patterns observed in past malaria outbreaks in Cabo Verde, which generally occurred in the post-rainy season [62]. In Praia, intense but infrequent rains may expand breeding sites [63], while seasonal changes can enhance *An. arabiensis* survival [64].

*Culex pipiens* s.l. were mostly present before the rainy season, suggesting greater effectiveness of the BGS trap in sampling this species during specific periods [65]. Several studies have linked *Cx. pipiens* abundance to warmer temperatures and reduced rainfall [66,67].

*Aedes aegypti* was the most abundant species captured across seasons, with collections occurring during both day and night periods. This finding indicates efficient environmental adaptation of this species in the Vale do Palmarejo neighborhood, reinforcing its establishment in the capital city, Praia [68], and raising concerns about the persistent risk of arbovirus transmission throughout the year.

With respect to gonotrophic state of captured females, most were unfed across all species, with an increased proportion of unfed females in BGS traps supplemented with CO_2_. MTego traps displayed a similar capture profile, also favoring unfed females. A notable abundance of gravid females of *Ae. aegypti* and *Cx. pipiens* s.l., as well as recently fed *An. arabiensis* was observed. These findings are relevant for designing control strategies targeting adult mosquitoes and suggest species-specific behavioral differences in the study area. Although extensive bionomic studies on *Ae. aegypti* populations in Cabo Verde have not yet been conducted, high ovitrap index (OI) values have been reported. Outdoor areas and water storage containers located outside households were identified as the principal breeding sites [17], suggesting preferential outdoor resting behavior. This exophilic tendency of *Ae. aegypti* has been documented in other African countries [69,70].

The results of this study provide valuable input for establishing a targeted high-precision entomological surveillance system in Cabo Verde in the context of malaria reintroduction. Differentiated use of traps, according to vector is recommended: MTego for *An. arabiensis* and BGS for *Ae. aegypti*. The creation of sentinel areas in strategic locations equipped with both trap types will facilitate monitoring of vector population dynamics across space and time, allowing targeted and timely interventions for vector control.

The relatively low number of Anopheles collected (n = 272), together with the pooling of *An. gambiae* s.l. species, constitutes an important limitation that may affect the precision and generalizability of trap‑performance estimates. This limitation reflects the naturally low and highly seasonal anopheline densities in Praia, where *An. arabiensis* is the only confirmed malaria vector. Nevertheless, the superior capture performance of MTego supplemented with CO_2_ was consistent throughout the sampling period, providing operationally relevant evidence for malaria surveillance in this setting. Future studies with larger sample sizes, species‑specific identification, and evaluations in indoor environments will be essential to enhance the robustness and broader applicability of these findings.

Conversely, the contribution of clay balls designed to generate moisture and thermal cues that mimic a human host after the addition of warm water [23,24] was not detectable under our experimental conditions. Because our design did not include MTego configurations that isolate these cues (e.g., without external CO_2_ and without clay balls, or with external CO_2_ but without clay balls), the specific effect of clay balls cannot be evaluated.

Our study has other limitations. Carbon dioxide (CO_2_) acts primarily as a long-range attractant; therefore, BGS traps, whether deployed alone or in combination with CO_2_, would benefit from supplementation with additional cues, such as heat, which functions as a short-range attractant [71,72]. Moreover, CO_2_ inflow from the bottles could not be controlled, as the homemade yeast–sugar fermentation system, adapted from Smallegange *et al*.’s (2010) method and applied in this low‑resource field context does not allow precise regulation of emission levels. In practical terms, this approach also entails operational constraints: preparation of the yeast–sugar mixture must be standardized across traps, and manual assembly of the delivery system requires additional field handling. Although this approach is widely adopted in community‑based surveillance programs due to its low cost and accessibility, future studies conducted under conditions with regulated CO_2_ sources are available should consider standardized delivery systems – such as compressed CO_2_ cylinders, controlled‑flow regulators, industrial fermentation modules, or synthetic CO_2_ mimics – to enable more accurate quantification of CO_2_ effects on trap performance. Additionally, during the second study period, following rainfall, four consecutive days of temperature and relative humidity records were lost due to a malfunction of the portable weather station. This interruption resulted in an incomplete dataset, limiting the robustness of subsequent comparative analyses across seasonal periods.

Finally, this study was conducted in a small geographical area – Vale do Palmarejo – with specific socio-environmental characteristics. Future larger-scale studies covering different locations in the city of Praia may yield more representative results on the performance of the two traps evaluated in Cabo Verde.

## Conclusions

MTego traps supplemented with CO_2_ bait represent an effective alternative for surveillance of the malaria vector *An. arabiensis* in Praia, Cabo Verde, and complement the BGS trap, which is routinely frequently used by the National Vector Control Program to monitor the primary dengue and Zika vector in the country, *Ae. aegypti*. The differences observed in the collections of these two mosquito species according to trap type reflect their distinct behavioural characteristics, with *An. arabiensis* predominantly captured outdoors after rainfall and at night, whereas *Ae. aegypti* captured during both daily periods before and after rainfall, with a preference for daytime activity.

Although the number of Anopheles collected was relatively low – reflecting the naturally sparse and seasonal anopheline populations in Praia – the consistently superior performance of MTego supplemented with CO_2_ throughout the sampling period provides operationally relevant evidence for malaria surveillance in this context. These findings are important for adapting the National Vector Control Program to the current scenario of malaria reintroduction and other VBDs in Cabo Verde. Future studies with larger sample sizes, species‑specific identification, and indoor evaluations are expected to further validate and expand the applicability of MTego and BGS traps as effective tools for integrated vector surveillance in Praia, Cabo Verde.

## Supporting information

S1 Data Temperature and relative humidity data of air collected during sampling. (XLSX)

S2 Data Complete mosquito sampling data. (XLSX)
